# Musculoskeletal manifestations in post-acute sequelae of SARS-CoV-2 infection: a systematic review and meta-analysis

**DOI:** 10.3389/fpubh.2025.1662953

**Published:** 2025-09-19

**Authors:** Amogh Verma, Sushma V. Naidu, Huma Sulthana, Aftab Ullah, Muhammed Shabil, Ranjana Sah, Rachana Mehta, Asif Jan, Nur Ul Ain, Abdur Rahim, Ursula Abu Nahla

**Affiliations:** ^1^Department of Internal Medicine, Rama Medical College Hospital and Research Centre, Hapur, Uttar Pradesh, India; ^2^Department of Pharmacology, PES University Institute of Medical Sciences and Research, Bangalore, India; ^3^Department of Pharmaceutical Chemistry, Faculty of Pharmacy, MS Ramaiah University of Applied Sciences, Bangalore, India; ^4^Department of Pharmacy, Abasyn University Peshawar, Peshawar, Khyber Pakhtunkhwa, Pakistan; ^5^Department of Pharmacy, University of Peshawar, Peshawar, Khyber Pakhtunkhwa, Pakistan; ^6^Department of Pharmacy Practice, Faculty of Pharmacy, MS Ramaiah University of Applied Sciences, Bangalore, India; ^7^Department of Paediatrics, Dr. D. Y. Patil Medical College Hospital and Research Centre, Dr. D. Y. Patil Vidyapeeth (Deemed-to-be-University), Pimpri, Pune, Maharashtra, India; ^8^Department of Public Health Dentistry, Dr. D. Y. Patil Dental College and Hospital, Dr. D. Y. Patil Vidyapeeth (Deemed-to-be-University), Pune, Maharashtra, India; ^9^Clinical Microbiology, RDC, Manav Rachna International Institute of Research and Studies, Faridabad, Haryana, India; ^10^Saidu Group of Teaching Hospitals, Saidu Sharif Swat, Khyber Pakhtunkhwa, Pakistan; ^11^District Headquarter Hospital Charsadda, Charsadda, Khyber Pakhtunkhwa, Pakistan; ^12^CECOS University of IT and Emerging Sciences, Peshawar, Pakistan; ^13^Faculty of Medicine, Hebron University, Hebron, West Bank, Palestine

**Keywords:** post-acute sequelae of SARS-CoV-2 infection, muscle pain, joint pain, muscle weakness, long COVID, good health and well being

## Abstract

**Background:**

The COVID-19 pandemic has highlighted a spectrum of long-term sequelae, with musculoskeletal symptoms being a substantial component of Post-Acute Sequelae of SARS-CoV-2 infection (PASC). This systematic review and meta-analysis aimed to evaluate the incidence and nature of musculoskeletal manifestations in individuals recovering from COVID-19.

**Methods:**

A systematic search across PubMed, Embase, and Web of Science was performed up to February 15, 2024, to identify studies reporting on musculoskeletal symptoms post-COVID-19. Observational studies which reported any musculoskeletal symptoms of PASC were included. Data were pooled using a random-effects model to calculate the incidence of symptoms, with subgroup analyses based on time since infection. Statistical analysis were conducted in R software (V 4.3).

**Results:**

Sixty-four studies were included, demonstrating a pooled prevalence of muscle pain at 28% (95% CI: 22%−35%), which increased to 25.9% (95% CI: 20.7%−31.7%) at 12 months post-infection. Joint pain showed a pooled prevalence of 14.8% (95% CI: 10.6%−20.2%), with no significant temporal change. Muscle weakness was observed in 12.9% (95% CI: 4.2%−32.9%) of patients. Notable heterogeneity was observed across studies (*I*^2^ > 89% for all symptoms).

**Conclusion:**

Musculoskeletal symptoms are prevalent in individuals with PASC, with muscle pain being the most common. The findings highlight the need for comprehensive clinical management and continuous research to create targeted treatments and revise care protocols as the pandemic evolves.

## Introduction

The COVID-19 pandemic, caused by the severe acute respiratory syndrome coronavirus 2 (SARS-CoV-2), has emerged as a defining global health crisis of the early 21st century (1, 2). Initially recognized for its acute respiratory symptoms, the disease spectrum of COVID-19 has since expanded to reveal a multifaceted impact on human health, challenging the medical community's understanding of viral infections (2). As the pandemic has progressed, it has become increasingly evident that COVID-19 is not merely a transient respiratory illness but a complex condition with the potential to cause persistent and multifarious health issues (3). Among these, musculoskeletal manifestations represent a significant and debilitating consequence for a considerable number of individuals recovering from the infection (4, 5).

Musculoskeletal symptoms, including muscle pain, joint pain, and muscle weakness have been documented with alarming frequency among patients in the post-acute phase of COVID-19 (6). These symptoms can persist for months beyond the initial infection, leading to a condition often called “long COVID” or post-acute sequelae of SARS-CoV-2 infection (PASC) (7). The persistence of such symptoms has profound implications for individuals' quality of life, ability to return to work, and overall functional status. Furthermore, the broad spectrum of severity, from mild discomfort to severe impairment, underscores the need for a deeper understanding of these manifestations to inform patient management and rehabilitation strategies (8–10). The exact mechanisms underlying the musculoskeletal manifestations of PASC remain incompletely understood, but emerging evidence suggests a complex interplay of inflammatory, immunological, and possibly vascular factors (11–13). This complexity is compounded by the heterogeneity of patient experiences, with some individuals recovering fully from their acute infection without sequelae while others endure long-term disabilities. The variability in patient outcomes highlights the importance of identifying the prevalence, risk factors, and potential pathophysiological mechanisms contributing to the persistence of musculoskeletal symptoms (14–16).

Given the global scale of the pandemic and the significant number of individuals affected by COVID-19, understanding the long-term consequences of the disease is critical. A systematic review and meta-analysis of the musculoskeletal manifestations of post-acute sequelae of COVID-19 provides an opportunity to synthesize available evidence, offering a clearer picture of these conditions' prevalence and characteristics. By elucidating the extent and nature of musculoskeletal manifestations in PASC, this review aims to assess the type and incidence of musculoskeletal manifestation of PASC.

## Methods

To investigate the musculoskeletal manifestations of the post-acute sequelae of COVID-19, a comprehensive systematic review and meta-analysis was conducted. The study protocol has been registered with PROSPERO, adhering to the preferred reporting items for systematic reviews and meta-analyses (PRISMA) guidelines (17) (Supplementary Table S1).

### Literature search

Initially, a detailed search strategy was developed to capture relevant studies published in several electronic databases, including PubMed, Embase, and Web of Science. The search was conducted using a combination of keywords and MeSH terms related to “COVID-19,” “SARS-CoV-2,” “musculoskeletal manifestations,” “post-acute sequelae,” and “long COVID.” To ensure a comprehensive retrieval of pertinent studies, no restrictions were placed on language, publication status, or study design. The search was carried out covering the period from the inception of each database until February 15 2024. The search strategy is given in Supplementary Table S2.

### Inclusion criteria

Studies were included if they reported on musculoskeletal symptoms in patients with post-acute sequelae of COVID-19. Observational studies of cross-sectional and cohort were included. Studies which reported acute symptoms of COVID-19 without reporting the PASC were excluded. Exclusion criteria encompassed studies that did not specifically address musculoskeletal outcomes, case reports, editorial comments, and reviews.

### Screening

Following the search, all identified records were imported into a Nested-Knowledge software, where duplicates were removed. Two independent reviewers then screened the titles and abstracts of the remaining records for eligibility, using predefined inclusion and exclusion criteria. Any discrepancies between the two reviewers were resolved with the help of a third reviewer.

### Data extraction

Eligible studies underwent a full-text review to confirm their suitability for inclusion in the meta-analysis. Data extraction was performed independently by two reviewers using a standardized form. The tagging function of Nested-Knowledge was used for extraction. Extracted information included study characteristics (e.g., author, year of publication, study design), participant demographics (e.g., age, gender), musculoskeletal manifestations reported. The quality of included studies was assessed using JBI tool.

### Statistical analysis

Meta-analysis was to pool data from studies reporting similar outcomes, using random-effects models to account for between-study heterogeneity. The prevalence of each type of musculoskeletal symptoms, along with 95% confidence intervals, was calculated. Heterogeneity among studies was quantified using the *I*^2^ statistic (18). Subgroup analyses were conducted based on the time point of assessment after getting initial COVID infection. Publication bias was assessed through Doi plots and LFK index (19). All statistical analyses were performed using a R software version 4.3 (20, 21). A 95% prediction interval was calculated to provide a range within which the true effect size is expected to lie in similar future studies.

## Results

### Literature search

**Figure 1:**
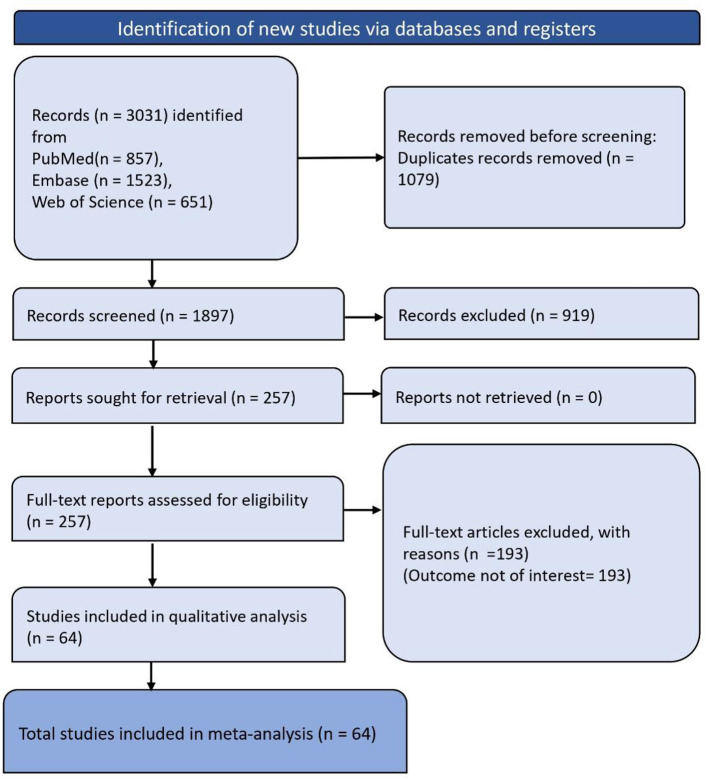
PRISMA flowchart depicting article screening and selection process.

The systematic review began with the identification of 3,031 records through database and registry searches, broken down as follows: 857 from PubMed, 1,523 from Embase, and 651 from Web of Science. Before screening, duplicates were removed, totaling 1,079 records. After deduplication, 1,897 records were screened, and subsequently, 919 of these records were excluded. The remaining 257 full-text reports were assessed for eligibility. Of these, 193 were excluded due to the outcome not being of interest. Finally, 64 studies were included in the meta-analysis (Figure 1).

### Characteristics of included studies

The summary of the included studies is given in Table 1. In total, 64 studies were encompassed in this synthesis, offering a broad overview of the clinical presentations associated with post-acute sequelae of COVID-19. These studies spanned numerous countries, with a majority conducted in 2023, indicating a concentrated effort to understand the long-term effects of the virus in recent times. The studies varied in design, with cohort studies being predominant, followed by cross-sectional studies, and a few retrospective cohort studies. The populations targeted were generally adult, with a few focusing on specific groups such as older adults with diabetes, health professionals, children, and patients with hypertension, showcasing the wide-reaching impact of COVID-19 across different demographic segments. Sample sizes across studies ranged from as few as 13 participants in a study involving football players in Italy to a significant cohort of 5,946 in Saudi Arabia, indicating the variance in the scale of these research efforts. The male percentage varied widely among studies, with some studies having a higher representation of male participants, like the study in Brazil with 70.9%, and others with a lower representation, such as 23.9% in a study from Saudi Arabia. Mean ages of participants spanned from as young as 12.1 years in a Turkish study on children to an older cohort with a mean age of 71 years in an Indian study, reflecting the diverse age range affected by post-COVID conditions. Musculoskeletal manifestations reported in these studies commonly included muscle pain and joint pain, with some studies also noting muscle weakness as a significant symptom. This indicates a consistent pattern of musculoskeletal issues in patients post-COVID infection, regardless of the country or population category. The quality assessment of studies is given in Supplementary Table S3.

**Table 1:** Characteristics of included studies.

**Author**	**Publication year**	**Country**	**Design**	**Population category**	**Total sample**	**Male %**	**Mean age**	**Musculoskeletal manifestations**
Al-Husinat (36)	2022	Jordan	Cross-sectional study	General	495	33.5	30.5	Muscle pain, muscle weakness
Alkwai (37)	2022	Saudi Arabia	Cross-sectional study	General	213	23.9	NA	Muscle weakness
Asadi-Pooya (38)	2021	Iran	Retrospective cohort study	General	2,685	50.9	52	Joint pain, muscle pain
Babicki (39)	2023	Poland	Cohort study	General	801	34.7	53.5	Joint pain, muscle pain
Bhandari (40)	2023	India	Cohort study	General	3,840	60.7	46.89	Muscle pain
Buttery (41)	2021	UK	Cross-sectional study	General	1,865	21.1	34.5	Muscle weakness
Chathoth (42)	2023	India	Cross-sectional study	General	938	56.5	41.5	Joint pain, muscle pain
Chudzik (43)	2022	Poland	Cohort study	General	218	31.0	45.74	Muscle pain
Dagher (44)	2023	USA	Cohort study	Cancer patients	188	NA	54.5	Muscle pain
Daitch (45)	2022	Israel, Switzerland, Spain, and Italy	Cohort study	Older adults	2,333	50.9	51.25	Joint pain, muscle pain
de Oliveira (46)	2022	Brazil	Cross-sectional study	General	369	50.3	58	Joint pain, muscle pain
di Filippo (47)	2023	Italy	Cross-sectional study	General	50	56	61.5	Joint pain, muscle pain
Duwel (48)	2023	Aruba	Retrospective cohort study	General	222	53.1	58.1	Joint pain, muscle pain
El Otmani (49)	2022	Morocco	Case control study	health professionals	118	28.8	33.25	Muscle pain
Emecen (50)	2023	Turkey	Cohort study	General	5,610	48	44.75	Muscle pain
Ercegovac (51)	2022	Serbia	Cross-sectional study	General	167	59.8	55.9	Joint pain
Fernández-de-Las-Peñas (52)	2022	Denmark	Cohort study	General	1,969	NA	61	Joint pain, muscle pain
Freire (53)	2022	Brazil	Cohort study	General	822	53.1	56	Muscle pain
Garout (54)	2022	Saudi Arabia	Cross-sectional study	General	744	49.2	33	Muscle pain
Gasnier (55)	2022	France	Cross-sectional study	General	177	40.5	57.2	Muscle weakness
Gattoni (56)	2022	Italy	Retrospective cohort study	Football players	13	100	23.9	Joint pain, muscle pain
Ghosn (57)	2023	France	Cohort study	General	737	64.4	61	Joint pain
Gonzalez-Aumatell (58)	2022	Spain	Cohort study	General	50	34	14.1	Joint pain, muscle pain, muscle weakness
Guadalupe Gutiérrez-Canales (59)	2022	Mexico	Cohort study	General	206	37.3	30.9	Joint pain, muscle pain
Hendrickson (60)	2023	USA	Cross-sectional study	General	284	33	45.2	Joint pain
Huang (61)	2022	China	Cohort study	General	1,192	53.7	56.75	Joint pain, muscle pain
Karaarslan (6)	2022	Turkey	Cohort study	General	291	59.4	52.54	Joint pain, muscle weakness
Karaarslan (62)	2021	Turkey	Cohort study	General	300	59.6	52.58	Joint pain, muscle pain
Kayaaslan (63)	2021	Turkey	Cohort study	General	1,007	54	45	Muscle pain
Kenny (64)	2022	Ireland	Cohort study	General	233	25.7	44	Joint pain, muscle pain
Magnavita (65)	2023	Italy	Cross-sectional study	Occupational cohorts	164	25	48.71	Muscle pain
Martino (66)	2022	Italy	Cohort study	General	64	64	66.75	Joint pain
Mateu (67)	2023	Spain	Cohort study	General	341	30.2	47.9	Joint pain, muscle pain
Maestre-Muñiz (68)	2021	Spain	Cross-sectional study	General	543	50.6	65.1	Muscle pain, muscle weakness
Muñoz-Corona (69)	2022	Mexico	Cohort study	General	141	59.5	52.24	Joint pain, muscle pain
Naik (70)	2021	India	Cohort study	General	1,234	69.3	41.6	Muscle pain
Sathyamurthy (71)	2021	India	Cohort study	General	279	36	71	Muscle pain
Paradowska-Nowakowska (72)	2023	Poland	Cross-sectional study	General	471	42.8	63.94	Muscle pain
Polese (73)	2023	Brazil	Cohort study	General	31	70.9	53.6	Muscle pain
Rass (74)	2022	Germany	Cohort study	General	81	59.2	54.75	Muscle pain
Román-Montes (75)	2023	Mexico	Cross-sectional study	General	246	54.8	52.5	Muscle pain
Romero (76)	2023	Colombia	Cross-sectional study	General	1,047	37.2	46.25	Joint pain, muscle pain
Sansone (77)	2022	Italy	Cohort study	General	247	35.6	48.1	Joint pain, muscle pain
Seang (78)	2022	Brazil	Cohort study	General	31	70.9	53.6	Muscle pain
Senjam (79)	2022	India	Cross-sectional study	General	257	56.4	34.75	Joint pain, muscle pain
Serrano (80)	2023	Colombia	Retrospective cohort study	General	135	70.3	61.75	Muscle weakness, muscle pain
Shivani (81)	2022	Pakistan	Cohort study	General	4,638	53.7	43	Muscle pain
Soh (82)	2022	South Korea	Cross-sectional study	General	147	53	52	Joint pain, muscle pain
Sousa (83)	2023	Brazil	Cross-sectional study	Older adults with diabetes	54	29.6	68.2	Muscle pain
Sykes (84)	2023	UK	Cohort study	General	144	62.5	62	Muscle pain
Tajer (85)	2023	Latin America	Cross-sectional study	Health professionals	3,642	35.8	47.8	Muscle pain
Talhari (86)	2023	Brazil	Ambidirectional cohort study	General	190	41	41.5	Joint pain, muscle pain, muscle weakness
Tejerina (87)	2022	Spain	Cohort study	General	29	37.9	45.25	Muscle pain
Tleyjeh (88)	2022	Saudi Arabia	Cross-sectional study	General	5,946	64.4	35.75	Joint pain, muscle pain
Tracy (89)	2024	USA	Cohort study	General	52	42.3	57.9	Muscle weakness
Vaira (90)	2022	Italy	Cross-sectional study	General	431	23.6	38.4	Joint pain, muscle pain
Wan (91)	2023	Malaysia	Retrospective cohort study	General	452	54.2	23.75	Joint pain, muscle pain
Wang (92)	2023	China	Cross-sectional study	General	1,546	50	57.93	Muscle pain
Wieteska-Mia, (93)	2023	Poland	Cohort study	Hypertension patients	69	31.8	50	Muscle pain
Wong (94)	2023	China	Cross-sectional study	General	2,712	40	NA	Joint pain, muscle pain
Wose Kinge (95)	2022	South Africa	Cross-sectional study	Frontline workers	62	24.1	35.25	Joint pain, muscle pain
Yaksi (96)	2022	Turkey	Retrospective cohort study	General	86	45.3	65.7	Muscle pain
Yildirim Arslan (97)	2023	Turkey	Cohort study	children	200	33.3	12.1	Joint pain
Zayet (98)	2021	France	Retrospective cohort study	General	354	37	49.6	Joint pain, muscle pain

### Muscle pain

**Figure 2:**
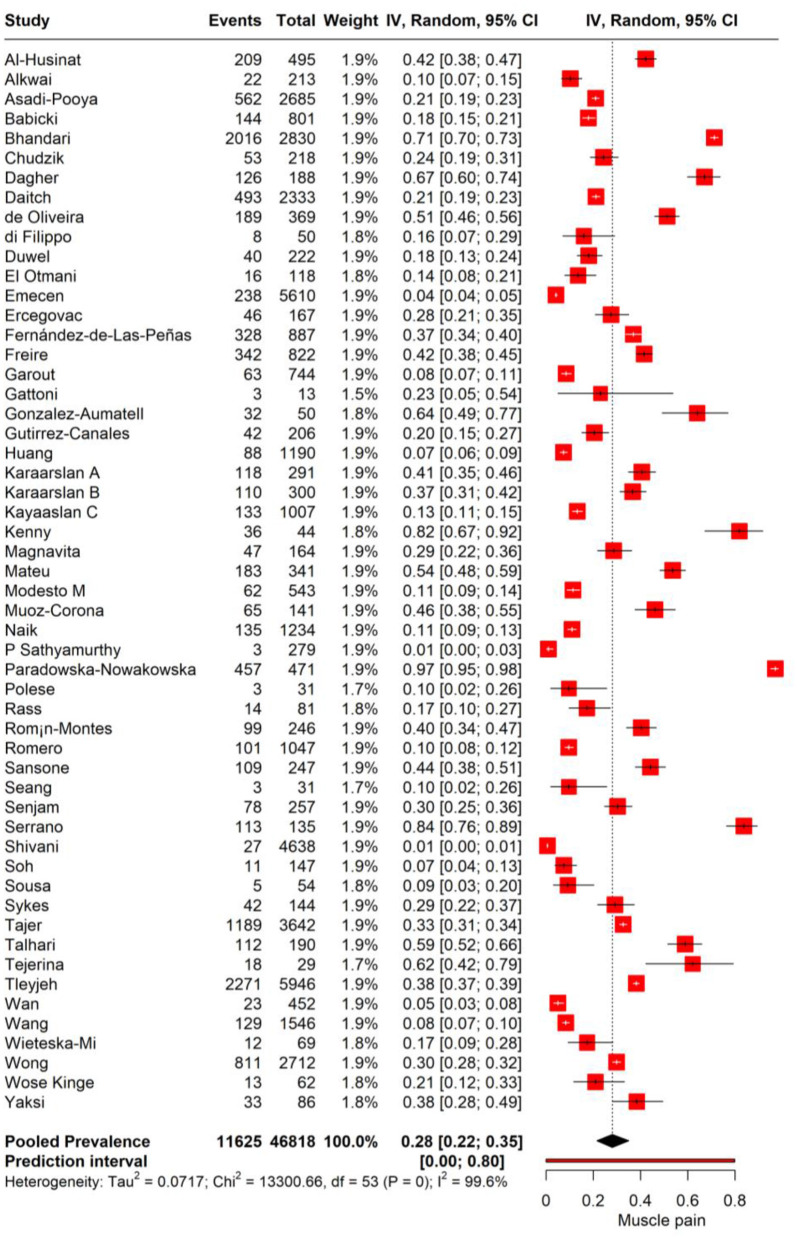
Forest plot showing pooled incidence of Muscle pain in PASC.

We performed meta-analysis to assess the incidence of muscle pain in individuals with PASC. The random-effects model yielded a pooled prevalence rate of 28% for muscle pain (95% CI: 22%−35%), with substantial heterogeneity (*I*^2^ = 100%, Tau^2^ = 0.0717). The prediction interval ranged expansively from 0.0 to 80%, suggesting that future research may encounter a similarly wide spectrum of muscle pain incidence among PASC patients (Figure 2).

**Figure 3:**
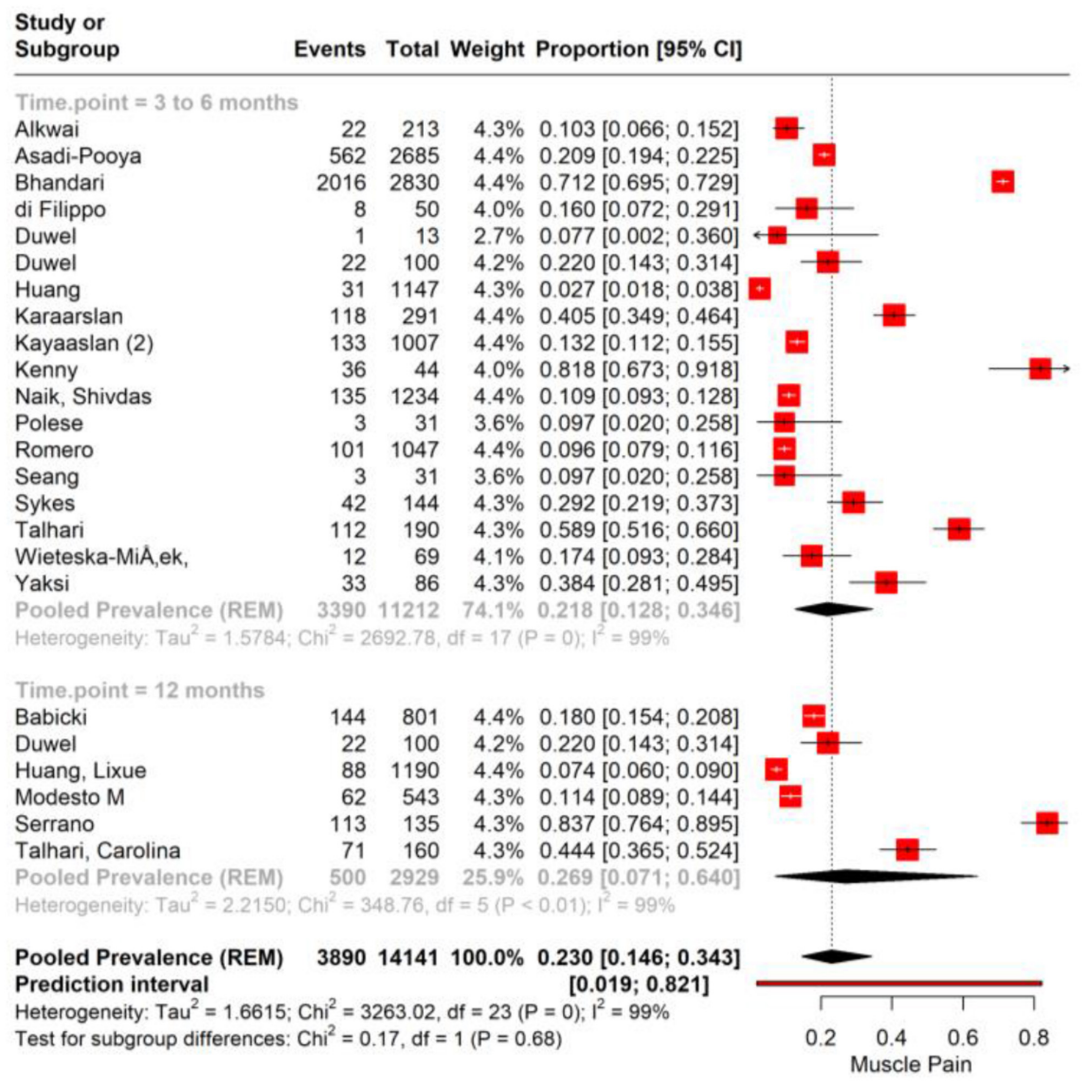
Forest plot showing pooled incidence of Muscle pain in PASC based on time point of assessment.

The subgroup analysis of muscle pain incidence in PASC based on different time points post-COVID-19 infection reveals distinct prevalence rates correlating with the duration post-infection. The analysis stratifies the data into two subgroups: the 3–6 months post-infection period and the point at 12 months post-infection. For the subgroup of 3–6 months post-infection, the meta-analysis reports a pooled prevalence of muscle pain at 17.4% (95% CI: 12.8%−22.1%). This suggests that within this time frame, on average, approximately one in six individuals may experience muscle pain as a symptom of PASC with high heterogeneity (*I*^2^ = 99.6%). In contrast, the subgroup representing 12 months post-infection demonstrates a pooled prevalence of 25.9% (95% CI: 20.7%−31.7%; Figure 3).

### Joint pain

**Figure 4:**
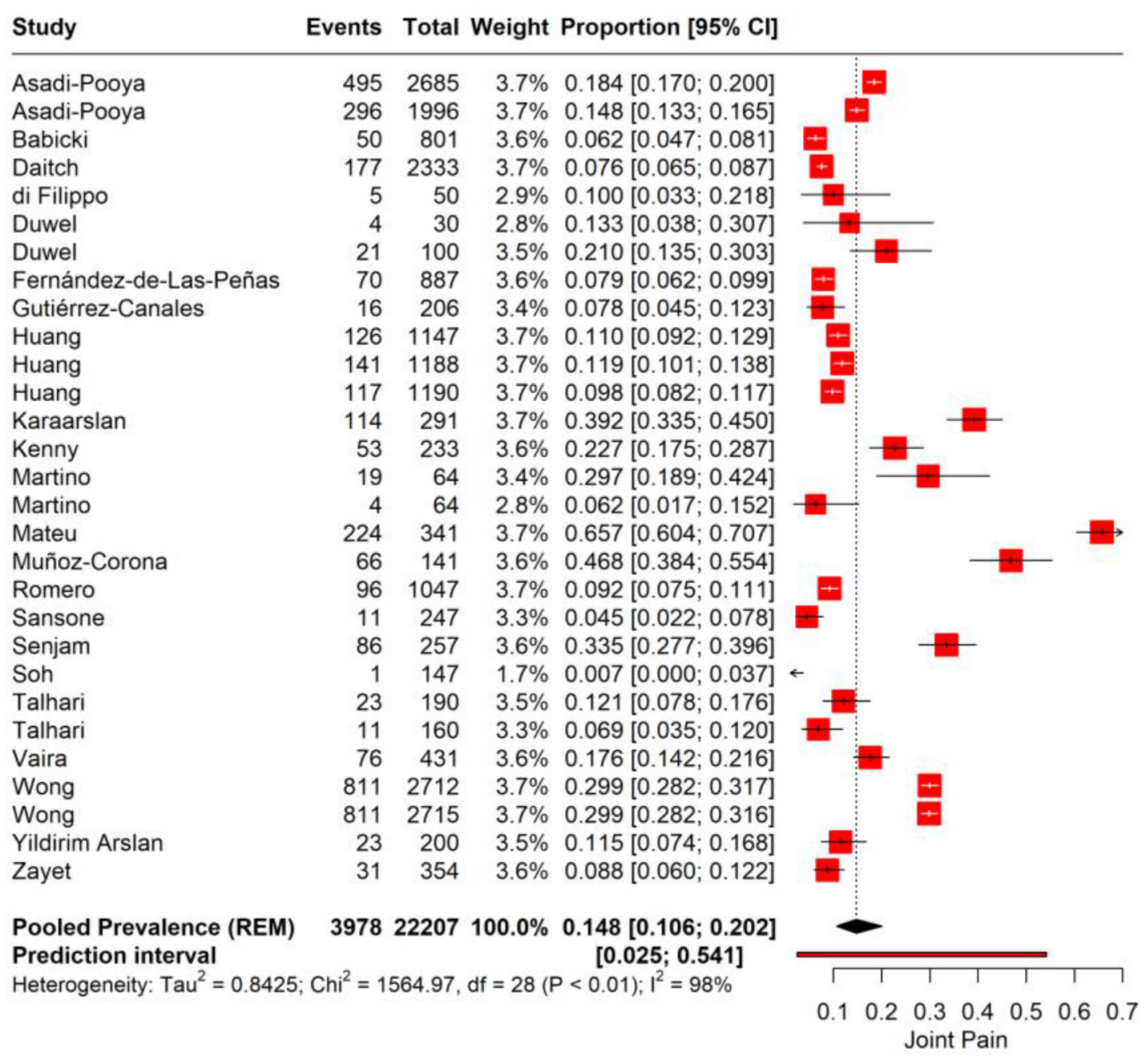
Forest plot showing pooled incidence of joint pain in PASC.

We performed a meta-analysis for joint pain incidence in PASC. The pooled prevalence rate for joint pain is observed at 14.8% (95% CI: 10.6%−20.2%), suggesting that on average, about one in seven individuals may experience joint pain as a sequela of COVID-19 infection. A high level of heterogeneity (*I*^2^ = 98%, Tau^2^ = 0.8425) among the studies was noted. The prediction interval, ranging from 2.5 to 54.1%, suggests that in a similar study context, the prevalence of joint pain could be expected to fall within this wide range (Figure 4).

**Figure 5:**
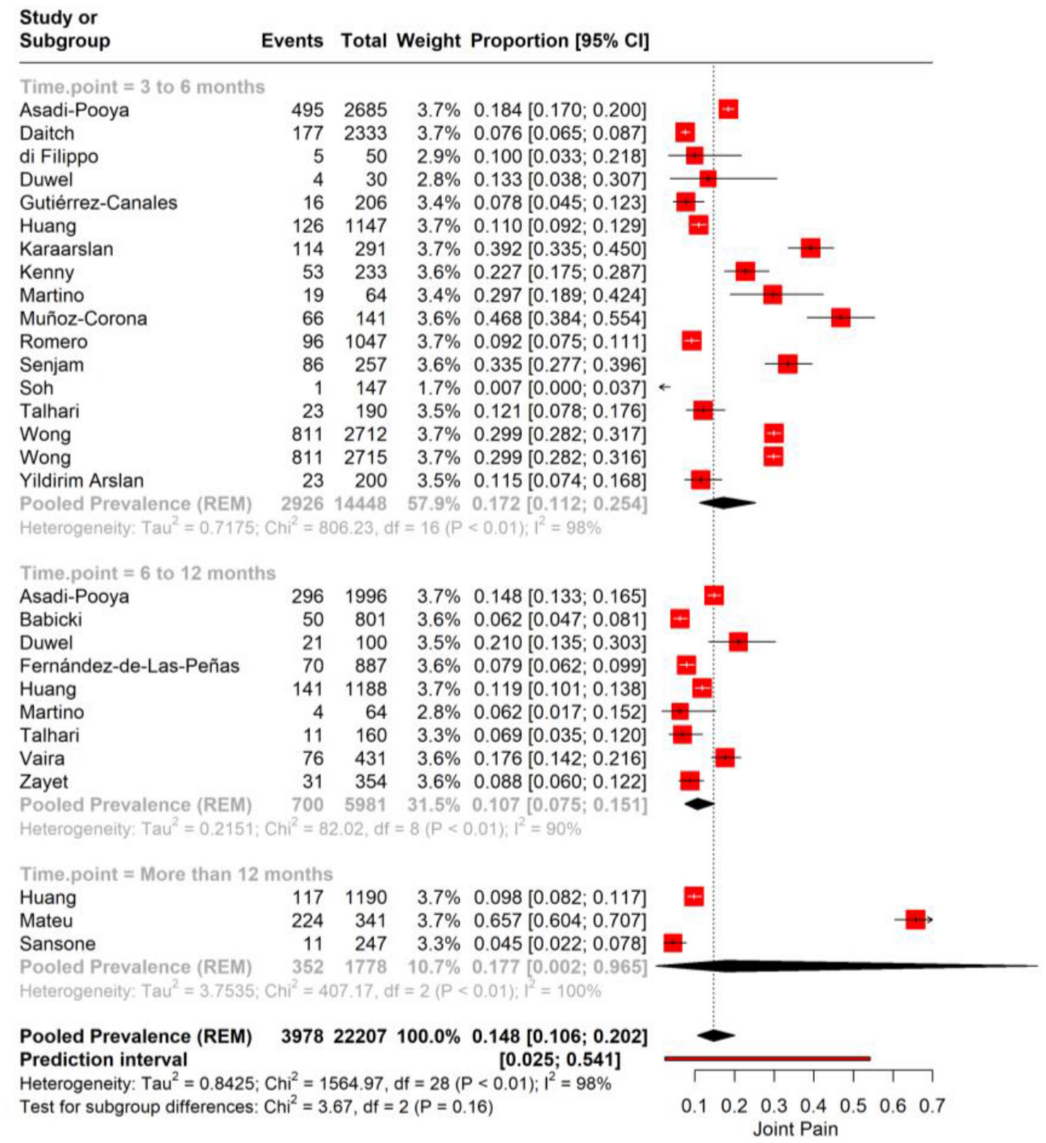
Forest plot showing pooled incidence of joint pain in PASC based on time point of assessment.

We performed subgroup analysis for joint pain based on the time point of assessment after the initial COVID-19 diagnosis. For the first subgroup, encompassing 3–6 months post-infection, the pooled prevalence of joint pain is 17.2% (95% CI: 12.1%−25.4%). The substantial heterogeneity observed (*I*^2^ = 89%) suggests significant variability in joint pain reporting across the included studies. In the 6–12 months post-infection subgroup, the data show a pooled prevalence of 10.7% (95% CI: 7.5%−15.1%) with high heterogeneity (*I*^2^ = 90; Figure 5).

### Muscle weakness

**Figure 6:**
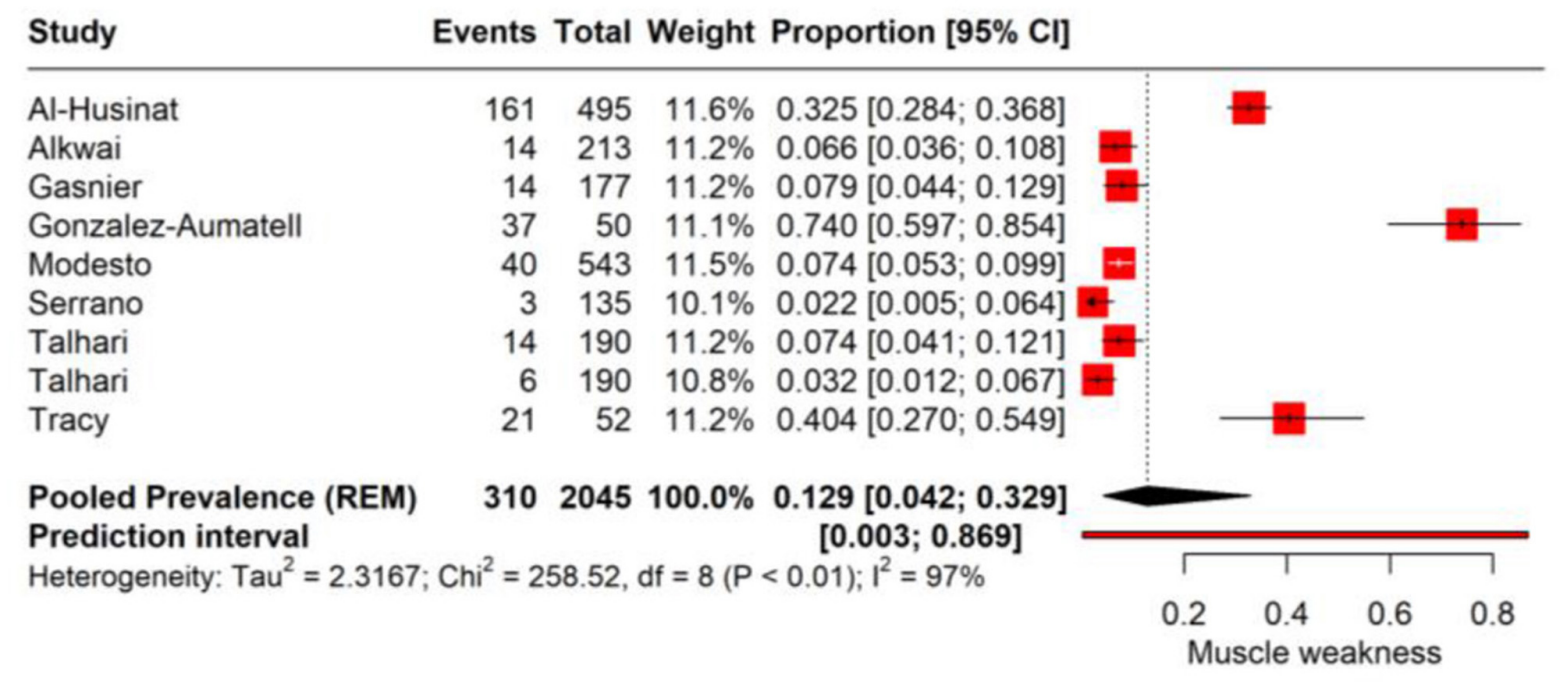
Forest plot showing pooled incidence of muscle weakness in PASC based on time point of assessment.

In the PASC, muscle weakness has been identified as a significant symptom affecting a considerable proportion of individuals. The pooled prevalence rate for muscle weakness is determined to be 12.9% (95% CI: 4.2%−32.9%, Tau^2^ = 2.31). This indicates that more than one in ten individuals may experience muscle weakness as a post-infection sequela, although there is a notable range in the confidence interval, suggesting variability in the symptom's manifestation. The heterogeneity present in the meta-analysis is significant, with an *I*^2^ value of 97%, reflecting considerable differences in the prevalence rates reported by the individual studies (Figure 6).

### Publication bias

**Figure 7:**
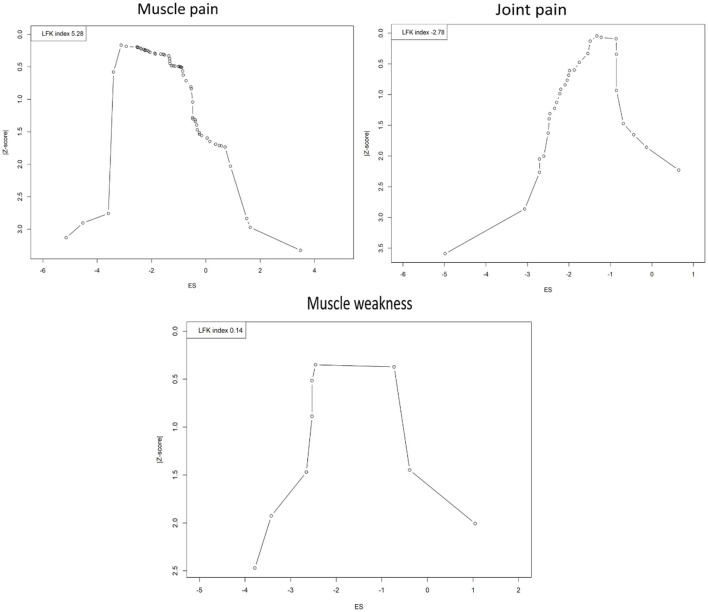
Doi plots showing assessment of publication bias.

The evaluation of publication bias across different musculoskeletal symptoms PASC of COVID-19 infection using Doi plots has produced varied results, each with distinct implications for the interpretation of meta-analytic data (Figure 7). For muscle pain, a pronounced asymmetry was detected with an LFK index of 5.28, substantially exceeding the threshold of 2 and suggesting the potential overrepresentation of studies with positive results. This could imply an overestimation of the actual effect size due to the underrepresentation of smaller or non-significant studies, thereby introducing a cautionary note in interpreting the pooled prevalence figures. Conversely, the analysis of joint pain studies indicated an asymmetry in the opposite direction with an LFK index of −2.78. This negative value may indicate a relative underestimation of effect sizes in smaller studies that have been published despite demonstrating weaker effects. Such asymmetry does not necessarily point to selective publication based on positive findings but suggests that conservative results from smaller studies are present in the literature. Nonetheless, the negative index highlights the need for careful interpretation and suggests the possibility of other biases or unaccounted heterogeneity influencing the results. The assessment for muscle weakness revealed an LFK index of 0.14, which falls within the acceptable range for symmetry and suggests no significant publication bias in the meta-analysis. The balanced representation of studies provides confidence in the pooled effect size estimate, supporting the reliability of the reported prevalence of muscle weakness.

### Meta-regression

We conducted a meta-regression analysis to examine the effects of moderators, including mean age, sample size, country, study design, and percentage of male participants, on the prevalence of diabetes in prison populations. No significant effects of these moderators were observed. The results of the meta-regression are presented in Supplementary Table S5.

## Discussion

This systematic review and meta-analysis have synthesized available evidence regarding the incidence of musculoskeletal symptoms in individuals with PASC. Our findings indicate that musculoskeletal manifestations, specifically muscle pain, joint pain, and muscle weakness, are prevalent symptoms experienced by a significant proportion of patients recovering from COVID-19. The pooled incidence of muscle pain was 24.1%, suggesting that nearly a quarter of individuals post-COVID-19 may experience this symptom. Interestingly, the incidence seemed to increase at 12 months post-infection, indicating a possibility of persisting or late-onset muscle pain in PASC. Joint pain was reported with a pooled prevalence of 14.8%, a less frequent but still significant symptom affecting patients in the long term. The lack of significant differences in prevalence between the 3–6 months and 6–12 months subgroups suggests that joint pain may manifest consistently over time post-infection. Muscle weakness, though less prevalent at 12.9%, is another notable symptom that may profoundly impact the functional recovery of individuals.

The clinical implications of these findings are profound. Healthcare providers managing the PASC of COVID-19 patients should be cognizant of the high likelihood of musculoskeletal symptoms, which can substantially hinder patients' recovery and quality of life (22, 23). The relatively high incidence of muscle pain and its increase over time underscores the need for pain management and rehabilitation strategies to be integrated into long COVID care protocols (24). Given the persistence of joint pain across time frames, clinicians should also consider long-term management plans for joint health, possibly incorporating anti-inflammatory treatments, physical therapy, and lifestyle modifications tailored to reduce pain and improve joint function. The impact of muscle weakness on functional ability may require targeted physical rehabilitation strategies, including strength training and occupational therapy, to assist patients in regaining their pre-infection levels of function and independence (25, 26). Understanding the scope of these symptoms can help in the allocation of appropriate resources for long COVID clinics and rehabilitation services. It may also inform public health messaging and patient education, preparing individuals for possible long-term sequelae following COVID-19 infection and emphasizing the importance of seeking care when needed. The development of specific guidelines for the assessment and management of PASC-associated musculoskeletal symptoms would be beneficial. Such guidelines would help standardize care, improve patient outcomes, and could be informed by ongoing research into the pathophysiological mechanisms underlying these persistent symptoms. Additionally, the psychological impact of chronic musculoskeletal pain should not be overlooked, and appropriate mental health support services should be made available to patients struggling with the long-term consequences of COVID-19 (27).

The high heterogeneity observed in our meta-analyses suggests substantial variability across studies, potentially limiting the generalizability of our pooled prevalence estimates for PASC. To explore this, we conducted a meta-regression analyzing moderators including mean age, gender distribution, geographic location, study design, and percentage of male participants, but no significant effects were identified. Data limitations precluded further analysis of other potential moderators. The high heterogeneity shows challenges in applying these findings to diverse populations or clinical guidelines. Future research should prioritize standardized reporting and larger, more diverse studies to better elucidate sources of variability and enhance the applicability of PASC prevalence estimates.

Future studies must prioritize longitudinal cohort designs that track the evolution of musculoskeletal symptoms from the acute phase of COVID-19 to the chronic phase, delineating their trajectory and identifying predictors of long-term disability. Concurrently, mechanistic studies should delve into the biological underpinnings of PASC, unraveling the inflammatory, immunological, and vascular factors that contribute to the persistence of musculoskeletal manifestations. The establishment of standardized diagnostic criteria will harmonize research efforts and enhance the comparability of findings across studies (28). Research should also appraise the effectiveness of multidisciplinary management strategies that integrate pharmacological and non-pharmacological interventions to support the holistic recovery of PASC patients (29). Investigating the health system's response to PASC will shed light on the efficacy of existing healthcare pathways, including the role of long COVID clinics and rehabilitation services (30, 31). Additionally, incorporating a global health perspective will ensure research is inclusive, capturing the experiences of diverse populations across varying socioeconomic and geographical contexts. As the COVID-19 landscape evolves with new variants and vaccination updates, it is crucial to integrate these changes into ongoing research (32). This will help assess their impact on the incidence, severity, and recovery trajectory of PASC, ensuring that findings remain relevant and responsive to the current state of the pandemic. The creation of registry databases for PASC can serve as a comprehensive repository for global data, aiding real-time analysis and informing public health policies (33–35). Research outcomes should be translated into public health initiatives and educational programs that empower patients and inform the broader community about the long-term consequences of COVID-19. By addressing these focused research priorities, we can better grasp the complexities of PASC and work toward more effective interventions and policies that alleviate the burden of long-term sequelae on individuals and healthcare systems. This forward-looking research agenda will facilitate a concerted and informed response to the ongoing challenges of the COVID-19 pandemic.

Our review has some limitations. The significant heterogeneity observed across may have impacted the pooled estimates. This may undermine the reliability and generalizability of our pooled prevalence estimates for specific clinical or public health applications. The potential for publication bias, particularly in studies reporting on muscle pain, introduces a degree of uncertainty into our findings. Despite conducting a meta-regression to explore potential moderators, including mean age, gender distribution, geographic location, study design, and percentage of male participants, no significant effects were identified, likely due to limited data availability. The temporal relationship between COVID-19 infection and the onset of musculoskeletal symptoms was also challenging to ascertain due to the reliance on self-reported data and the retrospective nature of many studies. These limitations, along with the lack of consistent reporting on COVID-19 severity and vaccination status, restrict our ability to fully elucidate symptom trajectories and may reduce the applicability of our findings to specific clinical or public health contexts.

The evolving nature of the COVID-19 pandemic, with new variants emerging and changing patterns of infection and immunity, may influence the incidence and presentation of PASC, including musculoskeletal manifestations. Therefore, these findings must be viewed as a snapshot in time, with the need for ongoing research to update and confirm these results as the pandemic continues to unfold.

## Conclusion

Musculoskeletal symptoms such as muscle pain, joint pain and muscle weakness are common in PASC. The persistent nature of these symptoms demands not only immediate clinical attention but also a sustained research effort to understand and mitigate their long-term impacts. As the pandemic evolves, continuous updating of clinical guidelines and patient management approaches will be essential to address the needs of those suffering from PASC.

## Data Availability

The raw data supporting the conclusions of this article will be made available by the authors, without undue reservation.
