# Supplementary material for: Musculoskeletal manifestations in post-acute sequelae of SARS-CoV-2 infection: a systematic review and meta-analysis
Source: Front Public Health. 2025 Sep 19;13:1662953. doi: 10.3389/fpubh.2025.1662953 (PMC12491175; doi:10.3389/fpubh.2025.1662953)
Supplement: Supplementary file 1 [file Table_1.docx]

**Supplementary Materials**

**Table S1.** PRISMA Checklist

**Table S2.** The adjusted search terms as per searched electronic databases

**Table S3.** Quality assessment of studies with the JBI tool

## **Table S1.** PRISMA Checklist

| **Section and Topic** | **Item #** | **Checklist item (Prevalence of kidney diseases among the dengue patients: A systematic review and meta-analysis)** | **Location where item is reported** |
| --- | --- | --- | --- |
| **TITLE** | | |  |
| Title | 1 | Identify the report as a systematic review. | 1 |
| **ABSTRACT** | | |  |
| Abstract | 2 | See the PRISMA 2020 for Abstracts checklist. (made as per the Journal guidelines) | 2 |
| **INTRODUCTION** | | |  |
| Rationale | 3 | Describe the rationale for the review in the context of existing knowledge. | 3 |
| Objectives | 4 | Provide an explicit statement of the objective(s) or question(s) the review addresses. | 3 |
| **METHODS** | | |  |
| Eligibility criteria | 5 | Specify the inclusion and exclusion criteria for the review and how studies were grouped for the syntheses. | 4 |
| Information sources | 6 | Specify all databases, registers, websites, organisations, reference lists and other sources searched or consulted to identify studies. Specify the date when each source was last searched or consulted. | 4, Table S2 |
| Search strategy | 7 | Present the full search strategies for all databases, registers and websites, including any filters and limits used. | Table S2 |
| Selection process | 8 | Specify the methods used to decide whether a study met the inclusion criteria of the review, including how many reviewers screened each record and each report retrieved, whether they worked independently, and if applicable, details of automation tools used in the process. | 4 |
| Data collection process | 9 | Specify the methods used to collect data from reports, including how many reviewers collected data from each report, whether they worked independently, any processes for obtaining or confirming data from study investigators, and if applicable, details of automation tools used in the process. | 4 |
| Data items | 10a | List and define all outcomes for which data were sought. Specify whether all results that were compatible with each outcome domain in each study were sought (e.g., for all measures, time points, analyses), and if not, the methods used to decide which results to collect. | 3 |
|  | 10b | List and define all other variables for which data were sought (e.g., participant and intervention characteristics, funding sources). Describe any assumptions made about any missing or unclear information. | 4, Table 1 |
| Study risk of bias assessment | 11 | Specify the methods used to assess risk of bias in the included studies, including details of the tool(s) used, how many reviewers assessed each study and whether they worked independently, and if applicable, details of automation tools used in the process. | Table S3 |
| Effect measures | 12 | Specify for each outcome the effect measure(s) (e.g. risk ratio, mean difference) used in the synthesis or presentation of results. | 5 |
| Synthesis methods | 13a | Describe the processes used to decide which studies were eligible for each synthesis (e.g. tabulating the study intervention characteristics and comparing against the planned groups for each synthesis (item #5)). | 4,5 |
|  | 13b | Describe any methods required to prepare the data for presentation or synthesis, such as handling of missing summary statistics, or data conversions. | NA |
|  | 13c | Describe any methods used to tabulate or visually display results of individual studies and syntheses. |  |
|  | 13d | Describe any methods used to synthesize results and provide a rationale for the choice(s). If meta-analysis was performed, describe the model(s), method(s) to identify the presence and extent of statistical heterogeneity, and software package(s) used. | 4.5 |
|  | 13e | Describe any methods used to explore possible causes of heterogeneity among study results (e.g. subgroup analysis, meta-regression). | 5 |
|  | 13f | Describe any sensitivity analyses conducted to assess robustness of the synthesized results. | NA |
| Reporting bias assessment | 14 | Describe any methods used to assess risk of bias due to missing results in a synthesis (arising from reporting biases). | 5 |
| Certainty assessment | 15 | Describe any methods used to assess certainty (or confidence) in the body of evidence for an outcome. | NA |
| **RESULTS** | | |  |
| Study selection | 16a | Describe the results of the search and selection process, from the number of records identified in the search to the number of studies included in the review, ideally using a flow diagram. | Figure 1 |
|  | 16b | Cite studies that might appear to meet the inclusion criteria, but which were excluded, and explain why they were excluded. | NA |
| Study characteristics | 17 | Cite each included study and present its characteristics. | 4,5 Table 1 |
| Risk of bias in studies | 18 | Present assessments of risk of bias for each included study. | Table S3 |
| Results of individual studies | 19 | For all outcomes, present, for each study: (a) summary statistics for each group (where appropriate) and (b) an effect estimate and its precision (e.g. confidence/credible interval), ideally using structured tables or plots. | Table 1, Figure 2 |
| Results of syntheses | 20a | For each synthesis, briefly summarise the characteristics and risk of bias among contributing studies. | 4 |
|  | 20b | Present results of all statistical syntheses conducted. If meta-analysis was done, present for each the summary estimate and its precision (e.g. confidence/credible interval) and measures of statistical heterogeneity. If comparing groups, describe the direction of the effect. | 5,4 Figure 2 |
|  | 20c | Present results of all investigations of possible causes of heterogeneity among study results. | 5, |
|  | 20d | Present results of all sensitivity analyses conducted to assess the robustness of the synthesized results. | NA |
| Reporting biases | 21 | Present assessments of risk of bias due to missing results (arising from reporting biases) for each synthesis assessed. |  |
| Certainty of evidence | 22 | Present assessments of certainty (or confidence) in the body of evidence for each outcome assessed. | NA |
| **DISCUSSION** | | |  |
| Discussion | 23a | Provide a general interpretation of the results in the context of other evidence. | 5,6, 7 |
|  | 23b | Discuss any limitations of the evidence included in the review. | 7 |
|  | 23c | Discuss any limitations of the review processes used. | 7 |
|  | 23d | Discuss implications of the results for practice, policy, and future research. | 7 |
| **OTHER INFORMATION** | | |  |
| Registration and protocol | 24a | Provide registration information for the review, including register name and registration number, or state that the review was not registered. | 3 |
|  | 24b | Indicate where the review protocol can be accessed, or state that a protocol was not prepared. | 3 |
|  | 24c | Describe and explain any amendments to information provided at registration or in the protocol. | NA |
| Support | 25 | Describe sources of financial or non-financial support for the review, and the role of the funders or sponsors in the review. | 8 |
| Competing interests | 26 | Declare any competing interests of review authors. | 7 |
| Availability of data, code and other materials | 27 | Report which of the following are publicly available and where they can be found: template data collection forms; data extracted from included studies; data used for all analyses; analytic code; any other materials used in the review. | Supplementary Materials |

**Table S2. The adjusted search terms as per searched electronic databases [as of 15.02.2024]**

| Database | Search Query | Results |
| --- | --- | --- |
| PubMed | "pasc"[All Fields] OR "post-acute sequelae"[All Fields]) AND ("COVID-19"[All Fields] OR "COVID"[All Fields] OR "SARS"[All Fields] OR "SARS-CoV-2"[All Fields])) OR ("Long COVID"[All Fields] OR "Long COVID-19"[All Fields] OR "chronic covid*"[All Fields] OR "Long-Haul COVID"[All Fields] OR "Post-COVID-19 Syndrome"[All Fields] OR "Post-Acute COVID-19"[All Fields])) AND ("Arthritis"[All Fields] OR "Osteoporosis"[All Fields] OR "Tendinitis"[All Fields] OR "Bursitis"[All Fields] OR "Fibromyalgia"[All Fields] OR "muscl*"[All Fields] OR "Ligament Sprain"[All Fields] OR "osteo*"[All Fields] OR "Scoliosis"[All Fields] OR "Kyphosis"[All Fields] OR "Lordosis"[All Fields] OR "Rhabdomyolysis"[All Fields] OR "Muscular"[All Fields] OR "Osteomalacia"[All Fields] OR "Bone"[All Fields] OR "Epicondylitis"[All Fields] OR "Golfer's Elbow"[All Fields] OR "Plantar Fasciitis"[All Fields] OR "arthralgia"[All Fields] OR "myalgia"[All Fields] OR "Fasciitis"[All Fields] OR "Osteomyelitis"[All Fields] OR "Polymyalgia Rheumatica"[All Fields] OR "Giant Cell Arteritis"[All Fields] OR "Systemic Sclerosis"[All Fields] OR "Scleroderma"[All Fields] OR "Myositis"[All Fields] OR "Osteonecrosis"[All Fields] OR "musculosk*"[All Fields]) | 857 |
| EMBASE | ((pasc:ab,ti OR 'post-acute sequelae':ab,ti) AND ('covid-19':ab,ti OR 'covid':ab,ti OR 'sars':ab,ti OR 'sars-cov-2':ab,ti) OR 'long covid':ab,ti OR 'long covid-19':ab,ti OR 'chronic covid*':ab,ti OR 'long-haul covid':ab,ti OR 'post-covid-19 syndrome':ab,ti OR 'post-acute covid-19':ab,ti) AND ('arthritis' OR 'osteoporosis' OR 'tendinitis' OR 'bursitis' OR 'fibromyalgia' OR 'muscl*' OR 'ligament sprain' OR 'osteo*' OR 'scoliosis' OR 'kyphosis' OR 'lordosis' OR 'rhabdomyolysis' OR 'muscular' OR 'osteomalacia' OR 'bone' OR 'epicondylitis' OR 'plantar fasciitis' OR 'arthralgia' OR 'myalgia' OR 'fasciitis' OR 'osteomyelitis' OR 'polymyalgia rheumatica' OR 'giant cell arteritis' OR 'systemic sclerosis' OR 'scleroderma' OR 'myositis' OR 'osteonecrosis' OR 'musculosk*') | 1523 |
| Web of science | ((PASC OR “post-acute sequelae”) AND ("COVID-19" OR "COVID" OR "SARS" OR “SARS-CoV-2”)) OR (“Long COVID” OR “Long COVID-19” OR “Chronic COVID*” OR “Long-Haul COVID” OR “Post-COVID-19 Syndrome” OR “Post-Acute COVID-19”) (Topic) AND ('Arthritis' OR 'Osteoporosis' OR 'Tendinitis' OR 'Bursitis' OR 'Fibromyalgia' OR 'muscl*' OR 'Ligament sprain' OR 'osteo*' OR 'Scoliosis' OR 'Kyphosis' OR 'Lordosis' OR 'Rhabdomyolysis' OR 'Muscular' OR 'Osteomalacia' OR 'Bone' OR 'Epicondylitis' OR 'Plantar fasciitis' OR 'arthralgia' OR 'myalgia' OR 'Fasciitis' OR 'Osteomyelitis' OR 'Polymyalgia rheumatica' OR 'Giant Cell Arteritis' OR 'Systemic Sclerosis' OR 'Scleroderma' OR 'Myositis' OR 'Osteonecrosis' OR 'musculosk*') (All Fields) | 651 |

**Table S4.** JBI tool for the quality assessment of studies

| Study | D1 | D2 | D3 | D4 | D5 | D6 | D7 | D8 | D9 | Overall Quality of the study |
| --- | --- | --- | --- | --- | --- | --- | --- | --- | --- | --- |
|  | Was the sample frame appropriate to address the target population? | Were study participants sampled appropriately? | Was the sample size adequate? | Were the study subjects and the setting described in detail? | Was the data analysis conducted with sufficient coverage of the identified sample? | Were valid methods used for the identification of the condition? | Was the condition measured in a standard, reliable way for all participants? | Was there an appropriate statistical analysis? | Was the response rate adequate, and if not, was the low response rate managed appropriately? |  |
| Al-Husinat (1) | Yes | Yes | Yes | Yes | Unclear | Yes | Unclear | Yes | Unclear | Moderate |
| Alkwai (2) | Yes | Yes | Yes | Yes | Yes | NA | Yes | Yes | Yes | High |
| Asadi-Pooya (3) | No | Yes | Yes | Yes | Yes | Yes | Unclear | Yes | Unclear | Low |
| Babicki(4) | Yes | No | Yes | Yes | Yes | Yes | Yes | Yes | Unclear | Moderate |
| Bhandari (5) | Yes | Yes | No | Yes | Yes | Yes | Yes | Unclear | Unclear | Moderate |
| Buttery (6) | Yes | Yes | Yes | Yes | Yes | Yes | Yes | Yes | Unclear | High |
| Chathoth (7) | Yes | Yes | Yes | Yes | Unclear | Yes | Yes | Yes | Unclear | Moderate |
| Chudzik (8) | Yes | Yes | Yes | Yes | Yes | Yes | Yes | Yes | Unclear | High |
| Dagher(9) | No | Yes | Yes | Yes | Unclear | Yes | Yes | Yes | Unclear | Moderate |
| Daitch (10) | Yes | No | Yes | Yes | Yes | Unclear | Yes | Yes | Yes | Low |
| de Oliveira (11) | Yes | Yes | Yes | Yes | Yes | Yes | Yes | Yes | Unclear | High |
| di Filippo, Luigi (12) | Yes | Yes | Yes | Yes | Unclear | Yes | Yes | Yes | Unclear | Moderate |
| Duwel, Veronika (13) | Yes | Yes | Yes | Yes | Yes | Yes | Unclear | Yes | Unclear | Moderate |
| El Otmani (14) | Yes | No | Yes | Yes | Yes | Yes | Yes | Yes | Unclear | Moderate |
| Emecen (15) | Yes | No |  | No | Yes | Yes | Yes | No | Unclear | Low |
| Ercegovac (16) | Yes | Yes | Yes | Yes | Yes | Yes | Yes | Yes | Unclear | High |
| Fernández-de-Las-Peñas (17) | Yes | Yes | Yes | Yes | Unclear | Yes | Yes | Yes | Unclear | Moderate |
| Freire (18) | Yes | Yes | Yes | Yes | Yes | Yes | Yes | Yes | Unclear | High |
| Garout (19) | Yes | Yes | Yes | Yes | Unclear | Yes | Yes | Yes | Unclear | Moderate |
| Gasnier (20) | Yes | No | Yes | Yes | Yes | Unclear | Yes | Yes | Yes | Moderate |
| Gattoni (21) | Yes | No | Yes | Yes | Yes | Yes | Yes | Yes | Unclear | Moderate |
| Ghosn (22) | Yes | Yes | No | Yes | Yes | Yes | Yes | Unclear | Unclear | Moderate |
| Gonzalez-Aumatell (23) | Yes | Yes | Yes | Yes | Yes | Yes | Yes | Yes | Unclear | High |
| Guadalupe Gutiérrez-Canales (24) | Yes | Yes | Yes | Yes | Unclear | Yes | Yes | Yes | Unclear | Moderate |
| Hendrickson (25) | No | No | Yes | Yes | Unclear | Yes | No | yes | Unclear | Low |
| Huang (26) | Yes | No | Yes | Yes | Yes | Yes | Yes | Yes | Unclear | Moderate |
| Karaarslan (27) | No | Yes | No | Yes | Yes | Yes | Yes | Unclear | Unclear | Moderate |
| Karaarslan (28) | Yes | Yes | Yes | Yes | Yes | Yes | Yes | Yes | Unclear | High |
| Kayaaslan (29) | Yes | Yes | Yes | Yes | Unclear | Yes | Yes | Yes | Unclear | Moderate |
| Kenny (30) | Yes | No | Yes | Yes | Yes | Unclear | Yes | Yes | Yes | Moderate |
| Magnavita (31) | Yes | No | Yes | Yes | Yes | Yes | Yes | Yes | Unclear | Moderate |
| Martino (32) | Yes | Yes | No | Yes | Yes | Yes | Yes | Unclear | Unclear | Moderate |
| Mateu (33) | Yes | Yes | Yes | Yes | Yes | Yes | Yes | Yes | Unclear | High |
| Modesto M (34) | Yes | Yes | Yes | Yes | Unclear | Yes | Yes | Yes | Unclear | Moderate |
| Muñoz-Corona (35) | Yes | Yes | Yes | Yes | Yes | Yes | Yes | Yes | Unclear | High |
| Naik (36) | Yes | Yes | Yes | Yes | Unclear | Yes | Yes | Yes | Unclear | Moderate |
| P, Sathyamurthy (37) | Yes | No | Yes | Yes | Yes | Unclear | Yes | Yes | Yes | Moderate |
| Paradowska-Nowakowska (38) | Yes | No | Yes | Yes | Yes | Yes | Yes | Yes | Unclear | Moderate |
| Polese (39) | No | Yes | Yes | Yes | Yes | Yes | Unclear | Yes | Unclear | Low |
| Rass (40) | Yes | No | Yes | Yes | Yes | Yes | Yes | Yes | Unclear | Moderate |
| Rom¡n-Montes (41) | Yes | Yes | No | Yes | Yes | Yes | Yes | Unclear | Unclear | Moderate |
| Romero (42) | Yes | Yes | Yes | Yes | Yes | Yes | Yes | Yes | Unclear | High |
| Sansone (43) | Yes | Yes | Yes | Yes | Unclear | Yes | Yes | Yes | Unclear | Moderate |
| Seang (44) | Yes | Yes | Yes | Yes | Yes | Yes | Yes | Yes | Unclear | High |
| Senjam (45) | Yes | No | Yes | Yes | Yes | Yes | Yes | Yes | Unclear | Moderate |
| Serrano (46) | Yes | Yes | No | Yes | Yes | Yes | Yes | Unclear | Unclear | Moderate |
| Shivani (47) | Yes | Yes | Yes | Yes | Yes | Yes | Yes | Yes | Unclear | High |
| Soh (48) | Yes | Yes | Yes | Yes | Unclear | Yes | Yes | Yes | Unclear | Moderate |
| Sousa (49) | Yes | Yes | Yes | Yes | Yes | Yes | Unclear | Yes | Unclear | Moderate |
| Sykes (50) | Yes | No | Yes | Yes | Yes | Yes | Yes | Yes | Unclear | Moderate |
| Tajer (51) | Yes | No |  | No | Yes | Yes | Yes | No | Unclear | Low |
| Talhari (52) | Yes | Yes | Yes | Yes | Yes | Yes | Yes | Yes | Unclear | High |
| Tejerina (53) | Yes | Yes | Yes | Yes | Unclear | Yes | Yes | Yes | Unclear | Moderate |
| Tleyjeh (54) | Yes | Yes | Yes | Yes | Yes | Yes | Unclear | Yes | Unclear | Moderate |
| Tracy (55) | No | Yes | No | Yes | Yes | Yes | Yes | Unclear | Unclear | Low |
| Vaira (56) | Yes | No | Yes | Yes | Yes | Unclear | Yes | Yes | Yes | Moderate |
| Wan (57) | Yes | Yes | Yes | Yes | Yes | Yes | Yes | Yes | Unclear | High |
| Wang (58) | Yes | Yes | Yes | Yes | Unclear | Yes | Yes | Yes | Unclear | Moderate |
| Wieteska-Mia‚ (59) | Yes | No | Yes | Yes | Yes | Unclear | Yes | Yes | Yes | Moderate |
| Wong (60) | Yes | No | Yes | Yes | Yes | Yes | Yes | Yes | Unclear | Moderate |
| Wose Kinge (61) | Yes | Yes | No | Yes | Yes | Yes | Yes | Unclear | Unclear | Moderate |
| Yaksi (62) | Yes | Yes | Yes | Yes | Yes | Yes | Yes | Yes | Unclear | High |
| Yildirim Arslan (63) | Yes | Yes | Yes | Yes | Unclear | Yes | Yes | Yes | Unclear | Moderate |
| Zayet (64) | Yes | Yes | Yes | Yes | Yes | Yes | Yes | Yes | Unclear | High |

| Moderator | Estimate | SE | Z-value | P-value | 95% CI Lower | 95% CI Upper |
| --- | --- | --- | --- | --- | --- | --- |
| Intercept | -193.3432 | 195.9750 | -0.9866 | 0.3239 | -577.4471 | 190.7607 |
| Country: Brazil | 0.3646 | 0.4385 | 0.8315 | 0.4057 | -0.4949 | 1.2241 |
| Country: China | 0.1392 | 0.4659 | 0.2988 | 0.7651 | -0.7740 | 1.0525 |
| Country: Colombia | 0.4058 | 0.4109 | 0.9874 | 0.3234 | -0.3997 | 1.2112 |
| Country: Germany | 0.3325 | 0.5329 | 0.6240 | 0.5326 | -0.7120 | 1.3770 |
| Country: India | 0.4365 | 0.4531 | 0.9634 | 0.3354 | -0.4515 | 1.3245 |
| Country: Iran | 0.2341 | 0.4890 | 0.4787 | 0.6321 | -0.7243 | 1.1925 |
| Country: Israel, Switzerland, Spain, and Italy | 0.3989 | 0.5208 | 0.7659 | 0.4438 | -0.6219 | 1.4197 |
| Country: Italy | 0.3588 | 0.4135 | 0.8678 | 0.3855 | -0.4516 | 1.1693 |
| Country: Jordan | 0.6506 | 0.5480 | 1.1872 | 0.2352 | -0.4235 | 1.7246 |
| Country: Latin America | 0.4446 | 0.5121 | 0.8681 | 0.3853 | -0.5592 | 1.4484 |
| Country: Malaysia | -0.2033 | 0.4847 | -0.4195 | 0.6748 | -1.1533 | 0.7466 |
| Country: Mexico | 0.5167 | 0.4490 | 1.1508 | 0.2498 | -0.3633 | 1.3968 |
| Country: Morocco | 0.0010 | 0.6340 | 0.0016 | 0.9987 | -1.2416 | 1.2436 |
| Country: Pakistan | -0.0067 | 0.5245 | -0.0127 | 0.9898 | -1.0346 | 1.0213 |
| Country: Poland | 0.5809 | 0.4239 | 1.3706 | 0.1705 | -0.2498 | 1.4117 |
| Country: Saudi Arabia | 0.3631 | 0.5035 | 0.7212 | 0.4708 | -0.6237 | 1.3500 |
| Country: Serbia | 0.4216 | 0.5402 | 0.7806 | 0.4351 | -0.6371 | 1.4803 |
| Country: South Africa | 0.4473 | 0.5462 | 0.8189 | 0.4129 | -0.6233 | 1.5178 |
| Country: South Korea | 0.1688 | 0.5337 | 0.3163 | 0.7518 | -0.8772 | 1.2148 |
| Country: Spain | 0.6926 | 0.4394 | 1.5762 | 0.1150 | -0.1686 | 1.5539 |
| Country: Turkey (1) | 0.3528 | 0.4170 | 0.8461 | 0.3975 | -0.4644 | 1.1700 |
| Country: UK | 0.3643 | 0.5388 | 0.6760 | 0.4990 | -0.6918 | 1.4203 |
| Design: Cohort study | -0.3641 | 0.3719 | -0.9790 | 0.3276 | -1.0929 | 0.3648 |
| Design: Cross-sectional study | -0.3350 | 0.3831 | -0.8745 | 0.3819 | -1.0858 | 0.4158 |
| Design: Retrospective cohort study | -0.1062 | 0.4768 | -0.2227 | 0.8237 | -1.0406 | 0.8282 |
| Male | 0.0026 | 0.0048 | 0.5385 | 0.5902 | -0.0068 | 0.0120 |
| Age | 0.0003 | 0.0056 | 0.0521 | 0.9584 | -0.0106 | 0.0112 |
| Year | 0.0958 | 0.0968 | 0.9891 | 0.3226 | -0.0940 | 0.2855 |

**Table S5: Meta-regression**

- Significance codes: * p < 0.05.
- Results are from a mixed-effects meta-regression model with 50 studies (k = 50) using REML estimation.
- Test of moderators: QM(df = 30) = 17.5704, p = 0.9651.
- Residual heterogeneity: tau² = 0.0985 (SE = 0.0327), tau = 0.3138, I² = 99.35%, H² = 153.31, R² = 0.00%.
- Test for residual heterogeneity: QE(df = 19) = 3124.1810, p < 0.0001.

1. Al-Husinat Li, Nusir M, Al-Gharaibeh H, Alomari AA, Smadi MM, Battaglini D, Pelosi P. Post-COVID-19 syndrome symptoms after mild and moderate SARS-CoV-2 infection. Frontiers in medicine. 2022;9:1017257.

2. Alkwai HM, Khalifa AM, Ahmed AM, Alnajib AM, Alshammari KA, Alrashidi MM, Ahmed HG. Persistence of COVID-19 symptoms beyond 3 months and the delayed return to the usual state of health in Saudi Arabia: A cross-sectional study. SAGE open medicine. 2022;10:20503121221129918.

3. Asadi-Pooya AA, Akbari A, Emami A, Lotfi M, Rostamihosseinkhani M, Nemati H, et al. Risk factors associated with long COVID syndrome: a retrospective study. Iranian journal of medical sciences. 2021;46(6):428.

4. Babicki M, Kapusta J, Pieniawska-Śmiech K, Kałuzińska-Kołat Ż, Kołat D, Mastalerz-Migas A, et al. Do COVID-19 Vaccinations Affect the Most Common Post-COVID Symptoms? Initial Data from the STOP-COVID Register–12-Month Follow-Up. Viruses. 2023;15(6):1370.

5. Bhandari S, Rankawat G, Joshi S, Tiwaskar M, Lohmror A. Post-COVID Syndrome: The Stranger Ghost of Culprit COVID-19. The Journal of the Association of Physicians of India. 2023;71(2):11-2.

6. Buttery S, Philip KE, Williams P, Fallas A, West B, Cumella A, et al. Patient symptoms and experience following COVID-19: results from a UK-wide survey. BMJ open respiratory research. 2021;8(1):e001075.

7. Chathoth AT, Anaswara N, Meethal AC, Vasudevan J, Gopal PV. Persisting and New Onset Symptomatology and Determinants of Functional Limitation of Post Acute COVID-19 Syndrome Cases-A Study from a Northern District of Kerala. Indian Journal of Community Medicine. 2023;48(2):250-7.

8. Chudzik M, Lewek J, Kapusta J, Banach M, Jankowski P, Bielecka-Dabrowa A. Predictors of long COVID in patients without comorbidities: data from the polish Long-COVID cardiovascular (PoLoCOV-CVD) study. Journal of Clinical Medicine. 2022;11(17):4980.

9. Dagher H, Chaftari A-M, Subbiah IM, Malek AE, Jiang Y, Lamie P, et al. Long COVID in cancer patients: preponderance of symptoms in majority of patients over long time period. Elife. 2023;12.

10. Daitch V, Yelin D, Awwad M, Guaraldi G, Milić J, Mussini C, et al. Characteristics of long-COVID among older adults: A cross-sectional study. International Journal of Infectious Diseases. 2022;125:287-93.

11. de Oliveira JF, de Ávila RE, de Oliveira NR, Sampaio NdCS, Botelho M, Gonçalves FA, et al. Persistent symptoms, quality of life, and risk factors in long COVID: a cross-sectional study of hospitalized patients in Brazil. International Journal of Infectious Diseases. 2022;122:1044-51.

12. di Filippo L, Frara S, Nannipieri F, Cotellessa A, Locatelli M, Rovere Querini P, Giustina A. Low vitamin D levels are associated with long COVID syndrome in COVID-19 survivors. The Journal of Clinical Endocrinology & Metabolism. 2023;108(10):e1106-e16.

13. Duwel V, de Kort JM, Becker CM, Kock SM, Tromp GG, Busari JO. A Cross-Sectional Study of the Physical and Mental Well-Being of Long COVID Patients in Aruba. Clinical Medicine & Research. 2023;21(2):69-78.

14. El Otmani H, Nabili S, Berrada M, Bellakhdar S, El Moutawakil B, Abdoh Rafai M. Prevalence, characteristics and risk factors in a Moroccan cohort of Long-Covid-19. Neurological Sciences. 2022;43(9):5175-80.

15. Emecen AN, Keskin S, Turunc O, Suner AF, Siyve N, Basoglu Sensoy E, et al. The presence of symptoms within 6 months after COVID-19: a single-center longitudinal study. Irish Journal of Medical Science (1971-). 2023;192(2):741-50.

16. Ercegovac M, Asanin M, Savic-Radojevic A, Ranin J, Matic M, Djukic T, et al. Antioxidant genetic profile modifies probability of developing neurological sequelae in long-COVID. Antioxidants. 2022;11(5):954.

17. Fernández-de-Las-Peñas C, de-la-Llave-Rincón AI, Ortega-Santiago R, Ambite-Quesada S, Gómez-Mayordomo V, Cuadrado ML, et al. Prevalence and risk factors of musculoskeletal pain symptoms as long-term post-COVID sequelae in hospitalized COVID-19 survivors: a multicenter study. Pain. 2022;163(9):e989-e96.

18. Freire MP, Oliveira MS, Magri MMC, Tavares BM, Marinho I, Nastri ACDSS, et al. Frequency and factors associated with hospital readmission after COVID-19 hospitalization: the importance of post-COVID diarrhea. Clinics. 2022;77:100061.

19. Garout MA, Saleh SA, Adly HM, Abdulkhaliq AA, Khafagy AA, Abdeltawab MR, et al. Post‐COVID-19 syndrome: assessment of short-and long-term post-recovery symptoms in recovered cases in Saudi Arabia. Infection. 2022;50(6):1431-9.

20. Gasnier M, Choucha W, Radiguer F, Faulet T, Chappell K, Bougarel A, et al. Comorbidity of long COVID and psychiatric disorders after a hospitalisation for COVID-19: a cross-sectional study. Journal of Neurology, Neurosurgery & Psychiatry. 2022;93(10):1091-8.

21. Gattoni C, Conti E, Casolo A, Nuccio S, Baglieri C, Capelli C, Girardi M. COVID‐19 disease in professional football players: symptoms and impact on pulmonary function and metabolic power during matches. Physiological Reports. 2022;10(11):e15337.

22. Ghosn J, Bachelet D, Livrozet M, Cervantes-Gonzalez M, Poissy J, Goehringer F, et al. Prevalence of post-acute coronavirus disease 2019 symptoms twelve months after hospitalization in participants retained in follow-up: analyses stratified by gender from a large prospective cohort. Clinical Microbiology and Infection. 2023;29(2):254. e7-. e13.

23. Gonzalez-Aumatell A, Bovo MV, Carreras-Abad C, Cuso-Perez S, Domènech Marsal È, Coll-Fernández R, et al. Social, academic and health status impact of long COVID on children and young people: an observational, descriptive, and longitudinal cohort study. Children. 2022;9(11):1677.

24. Gutiérrez-Canales LG, Muñoz-Corona C, Barrera-Chávez I, Viloria-Álvarez C, Macías AE, Guaní-Guerra E. Quality of Life and Persistence of Symptoms in Outpatients after Recovery from COVID-19. Medicina. 2022;58(12):1795.

25. Hendrickson KW, Hopkins RO, Groat DL, Stokes SC, Schroeder FM, Butler JM, Hirshberg EL. Patient experiences with SARS-CoV-2: Associations between patient experience of disease and coping profiles. Plos one. 2023;18(11):e0294201.

26. Huang L, Li X, Gu X, Zhang H, Ren L, Guo L, et al. Health outcomes in people 2 years after surviving hospitalisation with COVID-19: a longitudinal cohort study. The Lancet Respiratory Medicine. 2022;10(9):863-76.

27. Karaarslan F, Güneri FD, Kardeş S. Long COVID: rheumatologic/musculoskeletal symptoms in hospitalized COVID-19 survivors at 3 and 6 months. Clinical rheumatology. 2022;41:289-96.

28. Karaarslan F, Demircioğlu Güneri F, Kardeş S. Postdischarge rheumatic and musculoskeletal symptoms following hospitalization for COVID-19: prospective follow-up by phone interviews. Rheumatology international. 2021;41(7):1263-71.

29. Kayaaslan B, Eser F, Kalem AK, Kaya G, Kaplan B, Kacar D, et al. Post‐COVID syndrome: A single‐center questionnaire study on 1007 participants recovered from COVID‐19. Journal of medical virology. 2021;93(12):6566-74.

30. Kenny G, McCann K, O’Brien C, Savinelli S, Tinago W, Yousif O, et al., editors. Identification of distinct long COVID clinical phenotypes through cluster analysis of self-reported symptoms. Open forum infectious diseases; 2022: Oxford University Press US.

31. Magnavita N, Arnesano G, Di Prinzio RR, Gasbarri M, Meraglia I, Merella M, Vacca ME. Post-COVID symptoms in occupational cohorts: effects on health and work ability. International Journal of Environmental Research and Public Health. 2023;20(9):5638.

32. Martino GP, Benfaremo D, Bitti G, Valeri G, Postacchini L, Marchetti A, et al. 6 and 12 month outcomes in patients following COVID-19-related hospitalization: a prospective monocentric study. Internal and Emergency Medicine. 2022;17(6):1641-9.

33. Mateu L, Tebe C, Loste C, Santos JR, Lladós G, López C, et al. Determinants of the onset and prognosis of the post-COVID-19 condition: a 2-year prospective observational cohort study. The Lancet Regional Health–Europe. 2023;33.

34. Maestre-Muñiz MM, Arias Á, Mata-Vázquez E, Martín-Toledano M, López-Larramona G, Ruiz-Chicote AM, et al. Long-term outcomes of patients with coronavirus disease 2019 at one year after hospital discharge. Journal of clinical medicine. 2021;10(13):2945.

35. Muñoz-Corona C, Gutiérrez-Canales LG, Ortiz-Ledesma C, Martínez-Navarro LJ, Macías AE, Scavo-Montes DA, Guaní-Guerra E. Quality of life and persistence of COVID-19 symptoms 90 days after hospital discharge. Journal of International Medical Research. 2022;50(7):03000605221110492.

36. Naik S, Haldar SN, Soneja M, Mundadan NG, Garg P, Mittal A, et al. Post COVID-19 sequelae: A prospective observational study from Northern India. Drug discoveries & therapeutics. 2021;15(5):254-60.

37. Sathyamurthy P, Madhavan S, Pandurangan V. Prevalence, pattern and functional outcome of post COVID-19 syndrome in older adults. Cureus. 2021;13(8).

38. Paradowska-Nowakowska E, Łoboda D, Gołba KS, Sarecka-Hujar B. Long COVID-19 Syndrome Severity According to Sex, Time from the Onset of the Disease, and Exercise Capacity—The Results of a Cross-Sectional Study. Life. 2023;13(2):508.

39. Polese J, Ramos AD, Moulaz IR, Sant’Ana L, Lacerda BSdP, Soares CES, et al. Pulmonary function and exercise capacity six months after hospital discharge of patients with severe COVID-19. Brazilian Journal of Infectious Diseases. 2023;27:102789.

40. Rass V, Beer R, Schiefecker AJ, Lindner A, Kofler M, Ianosi BA, et al. Neurological outcomes 1 year after COVID‐19 diagnosis: A prospective longitudinal cohort study. European journal of neurology. 2022;29(6):1685-96.

41. Román-Montes CM, Flores-Soto Y, Guaracha-Basañez GA, Tamez-Torres KM, Sifuentes-Osornio J, González-Lara MF, León APd. Post-COVID-19 syndrome and quality of life impairment in severe COVID-19 Mexican patients. Frontiers in Public Health. 2023;11:1155951.

42. Romero M, Caicedo M, Díaz A, Ortega D, Llanos C, Concha A, et al. Post-COVID-19 syndrome: Descriptive analysis based on a survivors' cohort in Colombia. Global Epidemiology. 2023;6:100126.

43. Sansone D, Tassinari A, Valentinotti R, Kontogiannis D, Ronchese F, Centonze S, et al. Persistence of symptoms 15 months since COVID-19 diagnosis: prevalence, risk factors and residual work ability. Life. 2022;13(1):97.

44. Seang S, Itani O, Monsel G, Abdi B, Marcelin A, Valantin M, et al. Long COVID-19 symptoms: Clinical characteristics and recovery rate among non-severe outpatients over a six-month follow-up. Infectious Diseases Now. 2022;52(3):165-9.

45. Senjam SS, Balhara YPS, Kumar P, Nischal N, Manna S, Madan K, et al. A comprehensive assessment of self-reported post COVID-19 symptoms among beneficiaries of hospital employee scheme at a tertiary healthcare institution in Northern India. International Journal of General Medicine. 2022;15:7355.

46. Serrano MN, Muñoz OM, Rueda C, Arboleda AC-, Botero JD, Bustos MM. Factors associated with oxygen requirement and persistent symptoms 1 year after severe COVID-19 infection. Journal of International Medical Research. 2023;51(5):03000605231173317.

47. Shivani F, Kumari N, Bai P, Rakesh F, Haseeb M, Kumar S, et al. Long-term symptoms of COVID-19: One-year follow-up study. Cureus. 2022;14(6).

48. Soh HS, Cho B. Long COVID-19 and health-related quality of life of mild cases in Korea: 3-months follow-up of a single community treatment center. Journal of Korean medical science. 2022;37(46).

49. Sousa F, de Araujo LN, de Oliveira TSO, Gomes MC, Ferreira G, Aben-Athar C, et al. Demographic, Clinical, and Quality of Life Profiles of Older People With Diabetes During the COVID-19 Pandemic: Cross-Sectional Study. JMIR Formative Research. 2023;7(1):e49817.

50. Sykes DL, Van der Feltz‐Cornelis CM, Holdsworth L, Hart SP, O'Halloran J, Holding S, Crooks MG. Examining the relationship between inflammatory biomarkers during COVID‐19 hospitalization and subsequent long‐COVID symptoms: A longitudinal and retrospective study. Immunity, inflammation and disease. 2023;11(10):e1052.

51. Tajer C, JOSÉ M, Mariani J, De Abreu M, Antonietti L. Post COVID-19 syndrome. Severity and evolution in 4673 health care workers. MEDICINA (Buenos Aires). 2023;83:669-82.

52. Talhari C, Criado PR, Castro C, Ianhez M, Ramos PM, Miot HA. Prevalence of and risk factors for post-COVID: Results from a survey of 6,958 patients from Brazil. Anais da Academia Brasileira de Ciências. 2023;95:e20220143.

53. Tejerina F, Catalan P, Rodriguez-Grande C, Adan J, Rodriguez-Gonzalez C, Muñoz P, et al. Post-COVID-19 syndrome. SARS-CoV-2 RNA detection in plasma, stool, and urine in patients with persistent symptoms after COVID-19. BMC Infectious Diseases. 2022;22(1):211.

54. Tleyjeh IM, Kashour T, Riaz M, Amer SA, AlSwaidan N, Almutairi L, et al. Persistent COVID-19 symptoms at least one month after diagnosis: A national survey. Journal of infection and public health. 2022;15(5):578-85.

55. Tracy MF, Hagstrom S, Mathiason M, Wente S, Lindquist R. Emotional, mental health and physical symptom experience of patients hospitalized with COVID‐19 up to 3 months post‐hospitalization: A longitudinal study. Journal of Clinical Nursing. 2024;33(2):591-605.

56. Vaira LA, Gessa C, Deiana G, Salzano G, Maglitto F, Lechien JR, et al. The effects of persistent olfactory and gustatory dysfunctions on quality of life in long-COVID-19 patients. Life. 2022;12(2):141.

57. Wan KS, Sundram ER, Haddi AAA, Dashuki AR, Ahad A, John R, et al. Long COVID active case detection initiative among COVID-19 patients in Port Dickson, Malaysia: a retrospective study on the positive outcomes, the proportion of patients with long COVID and its associated factors. PeerJ. 2023;11:e14742.

58. Wang J-J, Zhang Q-F, Liu D, Du Q, Xu C, Wu Q-X, et al. Self-Reported Neurological Symptoms Two Years After Hospital Discharge Among COVID-19 Survivors. Journal of Alzheimer's Disease Reports. 2023(Preprint):1-6.

59. Wieteska‐Miłek M, Kuśmierczyk‐Droszcz B, Betkier‐Lipińska K, Szmit S, Florczyk M, Zieliński P, et al. Long COVID syndrome after SARS‐CoV‐2 survival in patients with pulmonary arterial hypertension and chronic thromboembolic pulmonary hypertension. Pulmonary Circulation. 2023;13(2):e12244.

60. Wong MC-S, Huang J, Wong Y-Y, Wong GL-H, Yip TC-F, Chan RN-Y, et al. Epidemiology, symptomatology, and risk factors for long COVID symptoms: population-based, multicenter study. JMIR Public Health and Surveillance. 2023;9(1):e42315.

61. Wose Kinge C, Hanekom S, Lupton-Smith A, Akpan F, Mothibi E, Maotoe T, et al. Persistent symptoms among frontline health workers post-acute COVID-19 infection. International Journal of Environmental Research and Public Health. 2022;19(10):5933.

62. Yaksi N, Teker AG, Imre A. Long COVID in hospitalized COVID-19 patients: A retrospective cohort study. Iranian Journal of Public Health. 2022;51(1):88.

63. Yildirim Arslan S, Avcu G, Sahbudak Bal Z, Arslan A, Ozkinay FF, Kurugol Z. Evaluation of post-COVID symptoms of the SARS-CoV-2 Delta and Omicron variants in children: A prospective study. European Journal of Pediatrics. 2023;182(10):4565-71.

64. Zayet S, Zahra H, Royer P-Y, Tipirdamaz C, Mercier J, Gendrin V, et al. Post-COVID-19 syndrome: nine months after SARS-CoV-2 infection in a cohort of 354 patients: data from the first wave of COVID-19 in Nord Franche-Comté Hospital, France. Microorganisms. 2021;9(8):1719.
